# Digestate-based fertilization supports lettuce growth with limited impacts on phyllosphere bacterial communities

**DOI:** 10.3389/fmicb.2026.1888705

**Published:** 2026-07-29

**Authors:** Camila Fabiani, M. Victoria Valero, Marianela Morales, Gastón A. Iocoli, Jessica Basualdo, Marco Allegrini, M. Bonita Villamil, M. Celina Zabaloy

**Affiliations:** 1 Consejo Nacional de Investigaciones Científicas y Técnicas (CONICET) Centro de Recursos Naturales Renovables de la Zona Semiárida (CERZOS), Departamento de Agronomía, Universidad Nacional del Sur, Bahía Blanca, Argentina; 2 Departamento de Agronomía, Universidad Nacional del Sur (UNS), Bahía Blanca, Argentina; 3 Consejo Nacional de Investigaciones Científicas y Técnicas (CONICET) Instituto de investigaciones Ciencias Agrarias Rosario (IICAR), Facultad de Ciencias Agrarias, Universidad Nacional de Rosario, Zavalla, Argentina; 4 Department of Crop Sciences, University of Illinois, Urbana, IL, United States

**Keywords:** anaerobic digestate, antibiotic resistance genes, digestate-based fertilization, fecal indicator bacteria, phyllosphere bacterial communities, plant performance, Salmonella

## Abstract

Digestate-based fertilizers are increasingly used in agriculture as part of circular nutrient management strategies, yet their effects on phyllosphere bacterial communities and antimicrobial resistance remain insufficiently understood. In this study, lettuce (*Lactuca sativa* L.) was grown under greenhouse conditions and subjected to different digestate-based fertilization strategies to evaluate their effects on plant performance, leaf nutrient content, cultivable fecal indicator bacteria and *Salmonella*, antibiotic resistance genes (ARGs), and phyllosphere bacterial communities. Microbiological quality was assessed through the analysis of coliforms and *Escherichia coli* by the most probable number (MPN) method and the detection of *Salmonella* spp. using standard culture-based procedures. ARG abundance was quantified by quantitative real-time PCR, while bacterial community composition was characterized using 16S rRNA gene sequencing and multivariate analyses. Notably, split application of digestate produced plant growth and leaf nutrient levels comparable to those achieved with inorganic fertilization, without increasing the abundance of the evaluated ARGs or the occurrence of fecal indicator bacteria and *Salmonella*, despite their detection in the initial digestate material. Fertilization strategy influenced phyllosphere bacterial community composition, whereas total bacterial abundance remained stable across treatments. Overall, these findings provide novel insights into digestate-based fertilization strategies by showing that, under the experimental conditions, digestate application supported lettuce productivity with limited impacts on phyllosphere bacterial communities.

## 1 Introduction

The growing demand for more sustainable agricultural practices has led to an increased use of organic amendments derived from waste materials, primarily to improve soil fertility while reducing environmental impacts and closing nutrient cycles. Among these amendments, anaerobic digestate is a by-product of the anaerobic digestion of various waste materials, including livestock manure. It is generated during biogas production, a process that converts organic waste into biomethane, thereby providing an alternative and renewable energy source. Rich in essential nutrients such as N, P, and K, digestate can serve as a soil amendment or fertilizer, representing a valuable agricultural resource that enhances soil quality (Iocoli et al., 2019), improve nutrient use efficiency, and contribute to environmentally sustainable production systems aligned with circular economy principles (Chojnacka and Moustakas, 2024; Martín-Sanz-Garrido et al., 2025). Nevertheless, its application involves potential microbiological and environmental concerns that require careful consideration (Fabiani et al., 2025; Zabaloy, 2021).

From a practical standpoint, digestate typically has a high moisture content (>90%), which makes its commercialization and long-distance transport unfeasible, increasing application costs and raising fossil fuel consumption. As a result, it is often applied to nearby fields, which can lead to nutrient overload and subsequent environmental contamination (Dhull et al., 2024). From a microbiological perspective, digestate contains a consortium of microorganisms and organic substrates that are introduced into the soil after application, potentially altering native microbial community composition and transiently modifying soil microbial activity through the addition of microbial biomass and readily available organic matter (van Midden et al., 2023). However, the effects of digestate on soil microbial community structure remain difficult to generalize because they strongly depend on digestate characteristics, feedstock composition, soil properties, and application rates. In addition, anaerobic digestion of manure carries the risk that residual antibiotics administered to livestock may persist in the excreta together with pathogenic microorganisms and antibiotic resistance genes (ARGs, Gurmessa et al., 2020). These ARGs can exist as intracellular DNA within resistant bacteria or as extracellular DNA associated with mobile genetic elements (MGEs) and can be transferred between bacteria in animal populations and from animals to humans, contributing to the global challenge of antimicrobial resistance (AMR), a serious public health threat (Allegrini and Zabaloy, 2024).

Macrolides, tetracyclines, and beta-lactams are widely used veterinary antibiotics whose resistance is associated with genes such as *ermB*, *tetW*, and *bla*_CTX_, respectively. These genes can persist in soils repeatedly amended with organic fertilizers (Blondeau, 2022). The dissemination of these ARGs is further facilitated by MGEs, including plasmids and class 1 integrons, which promote horizontal gene transfer among bacterial populations (Gillings, 2014).

Increasing evidence suggests that ARGs present in manure-amended soils can be transferred to plant-associated microbiomes through both internal and external pathways (Zhang et al., 2019). Internally, ARG-carrying microorganisms from the rhizosphere may colonize plant tissues through root uptake and subsequent upward translocation, reaching endophytic and phyllosphere communities. Externally, ARGs may also reach leaf surfaces through soil particles, aerosols, irrigation splash, or bioaerosols carrying resistant microorganisms (Chen et al.,2023; Zhang et al., 2019). Because microorganisms can move among the rhizosphere, endosphere, and phyllosphere, these plant compartments are interconnected and may share bacteria capable of harboring ARGs. Consequently, soil fertilization with manure-derived products may significantly alter not only soil microbial communities but also plant-associated bacterial communities (Barra Caracciolo et al., 2022; Chojnacka and Moustakas, 2024; Wolters et al., 2018). This is particularly relevant for leafy vegetables which are often consumed raw and, therefore, the phyllosphere represents a direct interface between agricultural practices and human health.

Despite growing interest in the agricultural use of anaerobic digestates, important knowledge gaps remain regarding their effects within the soil–plant-microbiome system. While several studies have focused on soil processes, far less attention has been paid to the potential transfer and persistence of ARGs, MGEs and bacterial pathogens onto the phyllosphere of food crops. Therefore, management strategies that mitigate the transfer of ARGs within the soil–plant system are of particular interest.

Among the strategies proposed to mitigate the environmental and microbiological risks associated with digestate application, combining digestate with pyrolytic biochar emerges as a promising approach. Biochar facilitates digestate drying, provides structural support, and enhances the retention of organic matter and nutrients (Ngigi et al., 2019), while also improving soil physicochemical and biological properties and influencing soil microbial communities (García-Prats et al., 2026). In addition, biochar may minimize the risk of resistance dissemination into the environment by reducing the abundance of potential ARGs host bacteria and promoting ARG adsorption through its high surface area and porous structure (García-Prats et al., 2026; Xu et al., 2022). Beyond reducing ARG abundance, biochar may also limit their horizontal transfer by removing MGEs (Jang and Kan, 2022; Ni et al., 2023).

In this context, we hypothesized that the application of anaerobic digestate introduces ARGs, MGEs, and potentially pathogenic microorganisms into the lettuce phyllosphere, while co-application with biochar mitigates their persistence by influencing microbial community structure and gene retention in soil. This study aims to generate applied knowledge on the use of manure-derived anaerobic digestates in agricultural systems, with a particular focus on plant-associated microbiomes of food crops. Specifically, the objectives are to (i) evaluate the effects of digestate-based fertilizers on lettuce growth and nutrient uptake, (ii) assess their impact on phyllosphere bacterial community composition, and (iii) quantify the abundance of selected ARGs, MGEs, and bacterial indicators associated with these fertilization strategies.

## 2 Materials and methods

### 2.1 Digestate obtention and preparation of the combined fertilizer

The anaerobic digestate used in this study was produced on-site at the BIODES plant in the locality of Coronel Suárez operated by Agro De Souza S. A., using two complete mix anaerobic reactors fueled entirely by feedlot cattle manure. The system features a European-style design and includes key components from Vogelsang^®^ (Germany). Anaerobic digestion is carried out under mesophilic conditions (39 °C), with a hydraulic retention time of 40 days. To produce the combined fertilizer, the digestate was acidified to pH 6.5 with nitric acid 65% and incorporated into the biochar (hardwood charcoal) with a predetermined ratio of 10.65 L of digestate per kg of biochar.

### 2.2 Digestate, biochar and combined fertilizer characterization

The following parameters were determined: NH_4_^+^-N, NO_3_^−^-N and total Kjeldahl nitrogen (TKN) by the semi-micro-Kjeldahl method. Total carbon (C) was determined by dry combustion (Elemental Analyser Exeter CE 440, Exeter Analytical UK Ltd., Coventry, England). Total solid (TS) was determined by drying samples at 105 °C to constant weight. The ash content was determined by burning samples at 550 °C for 2 h. The volatile solid (VS) was calculated by subtracting the ash mass from the TS mass. The determinations for total and available phosphorus (TP and AP, respectively) and heavy metals were carried out with an Inductively Coupled Plasma Optical Emission Spectrometer (ICP-OES), Shimadzu Simultaneous 9,000 (Shimadzu, Canby, OR, USA) (Supplementary Table S1). Stability measurements were performed using Residual Biogas Potential (RB) and Volatile Fatty Acids (VFA). The obtained values (RB = 0.107 L/g organic matter and VFA = 0.042 chemical oxygen demand/g organic matter) were within the regulatory limits (<0.25 and <0.43, respectively), confirming that the digestate was stable. Total coliforms, *Escherichia coli*, and *Salmonella* were determined in the initial digestate sample following the standard methodology specified in Argentine Resolution 19/2019 (Secretaría de Gobierno de Ambiente y Desarrollo Sustentable, 2019). In the initial anaerobic digestate sample, the total coliform count was 43 MPN/g fresh weight, with fecal coliforms and *E. coli* present at levels below detection (<3 MPN/g). However, the *Salmonella* count was 113 MPN/4 g fresh weight, which exceeded the limit set by the regulation for this pathogen (<3 MPN/4 g).

### 2.3 Greenhouse experiment: soil, plant material, treatments, and design

Plastic pots (3 L capacity) were filled with 3 kg of a clay loam Petrocalcic Hapludoll (USDA, 2022) with no prior history of organic amendments or animal grazing. Pre-germinated lettuce plants (*Lactuca sativa* L., curly-leaf variety) were transplanted into the pots, one plant per pot. The experiment consisted of eight treatments with four biological replicates each (32 pots in total). Pots were arranged randomly in the greenhouse and repositioned weekly to minimize spatial effects. The treatments were defined according to the fertilization source and application strategy (Table 1). Fertilization rates were standardized to provide the same total N dose per pot (58.33 mg kg^−1^, equivalent to ~140 kg N ha^−1^), calculated based on soil mineral N (total N: 0.22%, N-NH₄^+^: 26.66 ppm, N-NO₃^−^: 17.56 ppm) and crop requirements (Iocoli et al., 2019). Digestate and inorganic fertilizers were applied as aqueous solutions evenly distributed over the soil surface of each pot. Biochar and combined fertilizer were applied to the soil surface and lightly incorporated into the upper soil layer (0–5 cm) and supplemented with distilled water to equalize moisture conditions among pots. Application volumes were standardized to 50 mL per pot across all treatments.

**Table 1:** Description of fertilization treatments.

Treatment code	Fertilization source	Application regime	Application schedule (per pot)
C	Control: distilled water	Before transplanting *	50 ml distilled water
B	Biochar	Before transplanting	1.18 g
AD	Anaerobic digestate	Before transplanting	50 ml
ADS	Anaerobic digestate	Split**	3 DBT: 15 mL digestate + 35 mL water 20 DPT: 35 mL digestate + 15 mL water
CF	Combined fertilizer: anaerobic digestate + biochar	Before transplanting	4.61 g
CFS	Combined fertilizer: anaerobic digestate + biochar	Split	3 DBT: 1.4 g 20 DPT: 3.21 g
IF	Inorganic fertilizer: urea + monoammonium phosphate	Before transplanting	0.44 g urea
IFS	Inorganic fertilizer: urea + monoammonium phosphate	Split	3 DBT: 0.07 g MAP + 0.12 g urea 20 DPT: 0.32 g urea

Before transplanting: applied 3 days before transplanting (DBT). Split application: divided into two applications, with 30% applied at 3 DBT and the remaining 70% at 20 days post-transplanting (DPT), according to plant nutrient demand during its growth cycle.

### 2.4 Plant measurement and sampling

Once a week until harvest time, an aerial image of each plant was taken by placing the pots on a 0.25 m^2^ ring to subsequently calculate the coverage area using the CobCal v2.0 software developed by the Instituto Nacional de Tecnología Agropecuaria (INTA, Ferrari et al., 2008). At the time of harvest, 49 days post-transplanting, fresh weight (FW) and dry weight (DW, after oven-drying at 65 °C for 48 h) were measured. Leaf sections were preserved in sterile tubes (Zhu et al., 2017) and stored at 4 °C for analysis of culturable microorganisms and at −80 °C for subsequent extraction of metagenomic DNA from the phyllosphere.

### 2.5 Nutrient analysis

The analysis was performed in a commercial laboratory (Brookside Laboratories, Inc., New Bremen, OH, United States) using reference methods (Gavlak et al., 1994). Total N was determined by combustion using the Dumas method. Briefly, samples were combusted at ~900 °C in a resistance furnace under He/O₂ atmosphere, and N₂ in the combustion gases was quantified by thermal conductivity detection. The method has a detection limit of 0.10% N (dry basis) and an analytical reproducibility of ± 5%. Other macro- and micronutrients (P, S, B, K, Ca, Mg, Na, Al, Zn, Cu, Mn, Fe, and Mo) were determined following a nitric acid/hydrogen peroxide microwave digestion according to Sah and Miller (1992). Element concentrations were quantified by ICP-AES and/or AAS. Typical detection limits were ~0.01% for major elements (P, K, Ca, Mg) and ~0.2 mg kg^−1^ for trace elements (B, Zn, Cu, Fe, Mn, Mo), with a reproducibility of approximately ±7%.

### 2.6 Screening of fecal indicator bacteria and *Salmonella*

This screening was focused on treatments receiving digestate-derived inputs and the control due to their relevance for assessing potential food safety risks associated with digestate use. Total coliforms, *E. coli*, and *Salmonella* were determined using as a reference the Argentine Food Code (CAA) for the analysis of fresh leafy vegetables (Secretaría de Calidad en Salud Secretaría de Agricultura, Ganadería y Pesca, 2023). To estimate the concentration of coliforms in each sample, the Most Probable Number (MPN) method was used. Briefly, 20 g of leaf were added to a flask with 180 mL of peptone water (Britania Laboratories, Argentina) and shaken for 2 min to obtain the first dilution 1:10 (10^−1^). Decimal dilutions were prepared by adding 1 mL of the 1:10 dilution to a tube containing 9 mL of peptone water until reaching the 10^−3^ dilution. One ml of each prepared dilution was inoculated in triplicate into a tube with 9 mL of Lauryl Sulfate medium (Britania Laboratories, Argentina) with a Durham tube inside. Tubes were incubated at 37 °C (±2 °C) for 24 h. After incubation, the tubes were examined for gas production and turbidity. In cases of positive tubes, the protocol continues with tubes and plates with Mac Conkey media and agar, respectively (Britania Laboratories, Argentina). For screening of *Salmonella* spp., 20 g of leaf were added to a flask with 180 mL of Enrichment medium for Enterobacteria (Britania Laboratories, Argentina), shaken for 2 min and incubated at 35 °C for 24 ± 2 h. Thereafter, 0.1 mL of the culture was transferred into a tube with 10 mL of Rappaport Vassiliadis broth (RV, Britania Laboratories, Argentina) and 1 mL into a tube with 10 mL of Tetrathionate Broth (TT, Britania Laboratories, Argentina). The RV tubes were incubated for 24 ± 2 h at 42 ± 0.2 °C; and the TT tubes for 24 ± 2 h at 35 ± 2.0 °C. Then, 10 μL of each tube were streaked onto three different plates: with brilliant green agar (Britania Laboratories, Argentina), with Xylose Lysine Deoxycholate (Britania Laboratories, Argentina) agar and with Hektoen Enteric (Britania Laboratories, Argentina) agar and incubated at 35 °C for 24 ± 2 h.

### 2.7 DNA extraction and quality control

The extraction and purification of metagenomic DNA were performed using the DNA PuriPrep-Suelo Kit^®^ (INBIO Highway, Tandil, Argentina). For DNA extraction from the phyllosphere three randomly selected replicates of each treatment were used, therefore, all analyses derived from the DNA extraction were performed using three replicates per treatment (*n =* 3). Following the procedure described by Zhu et al. (2017) with minor modifications, 10 g of leaf (cut into 2 cm- pieces) were soaked in 40 mL of Phosphate Buffer (60 mM pH 7.6) with 10% Tween, left for 1 h under reciprocal shaking, filtered and then centrifuged for 15 min at 10,000 rpm (Heraeus Biofuge pico centrifuge, Kendro, Germany). The pellets were recovered, and the DNA PuriPrep-Suelo Kit^®^ protocol was followed. The obtained DNA was subsequently quantified using the QuantiFluor^®^ dsDNA System (Promega, Madison, WI, USA) in a Quantus fluorometer (Promega, Madison, WI, USA). The quality and integrity of the DNA were assessed from 1% agarose gel and absorbance ratios (260:230 and 260:280 nm ratio) in a DS-11 FX spectrophotometer (DeNovix Inc., Wilmington, DE, USA).

### 2.8 Analysis of total bacteria, resistance genes and integrons

To study the treatment effect on the total number of bacteria, a qPCR of the 16S rRNA gene was conducted. Antibiotic resistance in the samples was determined using previously optimized qPCR protocols evaluated in different environmental samples (Supplementary Table S2). Specifically, the genes *ermB*, *tetW*, *bla*_CTX_ group 1, and the integrons *intI1* and *aint1* associated with horizontal gene transfer were assessed (Supplementary Table S2). All amplifications, baseline corrections, melting curve analysis, and standard curve assessments were conducted in ABI 7500 Real-Time System and its associated software (7,500 Software v2.0.3, Applied Biosystems, Foster City, CA, USA). In all cases, the SYBR Green system was used. SYBR Green reactions were conducted using SsoAdvanced™ Universal SYBR^®^ Green Supermix (BioRad, California, USA), employing two different thermocycler programs. For 16S rRNA the protocol was as follows: 5 min of initial denaturation at 95 °C, and 35 cycles of 15 s denaturation at 95 °C, 30 s annealing (see temperature in Supplementary Table S2) and 45 s extension at 72 °C. For resistance genes and integrons 10 min of initial denaturation at 95 °C, and 40 cycles of 15 s denaturation at 95 °C, 20 s annealing (see temperatures in Supplementary Table S2) and 30 s extension at 72 °C. Standard curves were generated using of plasmids cotaining the appropiate inserts/fragments ranging from 10^2^ to 10^9^ gene copies μl^−1^. Amplification efficiencies ranged from 90 to 104%, with R^2^ values higher than 0.99 for all qPCR assays.

### 2.9 16S amplicon sequencing

The DNA samples were sequenced targeting the bacterial V4 region of the 16S rRNA gene using the Illumina MiSeq platform (Illumina, Inc., San Diego, CA, USA) with paired-end sequencing, yielding 250 nt reads. For amplification, the primers 515F (GTGYCAGCMGCCGCGGTAA) and 806R (GGACTACNVGGGTWTCTAAT) were used. The Fluidigm™ protocol was followed at the DNA Service Laboratory, Roy J. Carver Biotechnology Center, University of Illinois at Urbana-Champaign, USA. The FASTQ files were generated and demultiplexed by the sequencing service using bcl2fastq v2.20 Conversion Software (Illumina, San Diego, CA, USA).

### 2.10 Bioinformatics analysis

Sequence processing was performed in QIIME2 using the DADA2 algorithm for quality control, including primer removal, trimming, and chimera filtering. This resulted in high-quality amplicon sequence variants (ASVs). Trimming parameters were optimized to 283 bp for forward reads and 251 bp for reverse reads based on QIIME2’s Interactive Quality Plot tool, with an expected error threshold set to 2. ASVs were taxonomically classified using the SILVA 132 database (Yilmaz et al., 2014) through QIIME2’s feature-classifier plugin. In the bacterial dataset, ASVs classified as archaea, mitochondria, chloroplasts, or unassigned were removed prior to analysis, resulting in a final set of 311 bacterial ASVs. Alpha-diversity metrics, including observed richness (S = number of ASVs), Chao1 index, Shannon diversity index, reciprocal Simpson index, and Pielou’s evenness were calculated as in Allegrini et al. (2019) and Morales et al. (2024). To calculate beta-diversity metrics, representative ASV sequences were aligned using MAFFT v7 (Katoh et al., 2019) and then analyzed using the phangorn v2.5.5 (Schliep, 2011) package to construct a distance matrix and a neighbor-joining phylogenetic tree. The resulting phylogenetic tree and ASV table were then used as input for the GUniFrac package v1.9 (Chen et al., 2025) to calculate generalized UniFrac distances.

### 2.11 Statistical analyses

All statistical analyses were conducted in Rstudio (R Core Team, 2025). Differences in LA, FW and DW, alpha-diversity metrics, ARG abundance (qPCR data, copy number ng^−1^ DNA), and nutrient composition were evaluated using one-way ANOVA followed by Tukey’s post-hoc tests (*α* = 0.05). For variables that did not meet normality assumptions, the non-parametric Kruskal–Wallis test was applied, followed by Dunn’s post-hoc test with Bonferroni correction.

Amplicon sequencing data were analyzed in RStudio using both standard and compositional data analysis approaches (Gloor and Reid, 2016; Gloor et al., 2017). In the standard approach, UniFrac distances were used as input for multivariate analysis of beta-diversity through non-metric multidimensional scaling (NMDS) using the metaMDS function of the vegan package v2.5–7 (Oksanen et al., 2020). Using the same generalized UniFrac distance matrix, a complementary multivariate analysis was performed using PERMANOVA (α = 0.05, Anderson, 2001) with the adonis function from the vegan package (1,000 permutations) to evaluate the effects of fertilization on community structure. When appropriate, pairwise PERMANOVA tests were conducted using the pairwise.perm.manova function in the RVAideMemoire package v.0.9–80 with 1,000 permutations and *p*-values adjusted using the false discovery rate (FDR) method (Hervé, 2021).

For the compositional approach, analyses were conducted using the ASV table generated by the SILVA classification platform. ASVs present in fewer than 25% of the samples were removed to reduce the influence of rare or potentially spurious taxa (Botha et al., 2024), resulting in a dataset of 91 bacterial ASVs. To identify ASVs most responsive to the treatments, a bootstrap forest partitioning approach was implemented using the JMP^®^ predictor screening platform (Villamil et al., 2021). This analysis identified 39 bacterial ASVs as significant contributors (≥1% contribution). The resulting ASV table was processed with the zCompositions package v.1.3.4 (Palarea-Albaladejo and Martín-Fernández, 2015) to replace zero values prior to the centered log-ratio (clr) transformation (Aitchison, 1982). The clr-transformed data were then used as input for PCA based on singular value decomposition using the FactoMineR package v.2.4 (Lê et al., 2008). PCs with eigenvalues ≥1 and explaining at least 5% of the variability in the dataset were used as independent variables for further analysis. ASVs showing a strong correlation with each PC (PC loading value >|0.60|) were considered microbial indicators and used in the description of the PC. PC scores for the retained components were analyzed using one-way ANOVA with treatment as a fixed effect, followed by Tukey’s *post hoc* test (*α* = 0.05) using emmeans (Lenth, 2021).

## 3 Results

### 3.1 Plant growth parameters

Digestate-based fertilization significantly increased lettuce biomass compared with unfertilized control, with plant growth comparable to that observed under inorganic fertilization. For all evaluated parameters-LA, FW, and DW- significant differences were observed among treatments (Table 2). The highest FW and DW were recorded in the IFS treatment (158.94 g and 10.37 g, respectively), which also exhibited the largest LA (1,249 cm^2^).

**Table 2:** Mean fresh weight (FW), dry weight (DW), and leaf area (LA) of plants under different fertilization treatments.

Treatment	*n*	FW (g)		DW (g)		LA (cm^2^)	
		Mean	S. E.	Mean	S. E.	Mean	S. E.
C	4	46.86 ^a^	9.11	5.38 ^b^	9.87	634 ^a^	46.7
B	4	39.48 ^a^	9.11	3.14 ^a^	9.87	594 ^a^	46.7
AD	4	111.36 ^cd^	9.11	8.82 ^cd^	9.87	1,022 ^bc^	46.7
ADS	4	113.87 ^cd^	9.11	7.22 ^bc^	9.87	1,131 ^cd^	46.7
CF	4	80.22 ^b^	9.11	7.69 ^c^	9.87	987 ^bc^	46.7
CFS	4	88.91 ^bc^	9.11	5.42 ^b^	9.87	915 ^b^	46.7
IF	4	116.29 ^d^	9.11	8.88 ^cd^	9.87	1,053 ^c^	46.7
IFS	4	158.94 ^e^	9.11	10.37 ^d^	9.87	1,249 ^d^	46.7
*F- value*		18.63		9.87		24.06	
df *		7,24		7,24		7,24	
*p*-value		<0.001		<0.001		<0.001	

Different letters within each column indicate significant differences between treatments (Tukey’s, *p <* 0.001). *F*-values, degrees of freedom (*df*; factor, error) and *p*-values are shown.

Plants treated with inorganic fertilizer in a single application (IF), as well as those treated with digestate (AD and ADS) or combined fertilizer (CF and CFS), showed significantly higher FW compared to C and B treatments. These two treatments consistently exhibited the lowest values, which aligned with their reduced LA (Table 2).

Regarding DW, plants treated with B had the lowest values, followed by those in the control group, which did not differ significantly from the split applications of digestate-based fertilizers (ADS and CFS). In contrast, single applications of digestate-based fertilizers (AD and CF) resulted in higher DW than their fractioned counterparts, reaching values comparable to those obtained with inorganic fertilizer (Table 2).

### 3.2 Nutrient content

**Figure 1:**
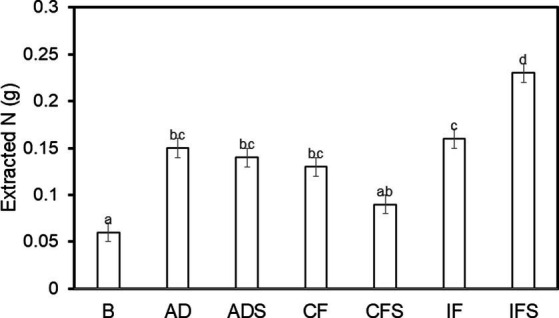
Extracted N by lettuce plants under the different fertilization treatments, calculated as biomass [DW]*%N. Bars represent mean ± S. E. (*n =* 4). Different letters indicate significant differences among treatments (Tukey’s, *p <* 0.05).

The content of 13 macro- and micronutrients (N, P, K, Ca, Mg, S, B, Fe, Mn, Cu, Zn, Al, and Na) were measured in lettuce plants under different treatments. The unfertilized control was not included in the nutrient analysis due to insufficient dry biomass. One-way ANOVA revealed significant differences among treatments for N (*p* = 0.0038), K (*p* = 0.0002), S (*p* = 0.008), Cu (*p* = 0.027, although *post hoc* tests did not separate treatments into distinct groups), and Mn (*p* = 0.028), whereas the other elements showed no significant differences (Table 3; Supplementary Table S3). For nutrients that showed a significant response to fertilization, IFS exhibited the highest concentrations, presenting the highest values of N, K, and Mn, and high S content. Notably, values obtained after fertilization with ADS were frequently comparable to those of IFS, with no statistically significant differences detected (Table 3). Based on tissue N content and biomass (DW), the extracted N for each treatment was calculated. It differed significantly among treatments (*p <* 0.0001). IFS showed the highest N extraction, while the IF and the digestate treatments alone or combined (CF) showed similar extraction values among them, almost 3 times higher than for B (Figure 1).

**Table 3:** Content of N, K, S and Mn in lettuce plants grown under the different fertilization treatments.

Treatment^†^	*n*	N (%)		K (%)		S (%)		Mn (ppm)	
		Mean	S. E.	Mean	S. E.	Mean	S. E.	Mean	S. E.
B	4	1.80 ^ab^	0.10	4.76 ^ab^	0.25	0.29 ^b^	0.02	1.95 ^ab^	0.07
AD	4	1.68 ^a^	0.10	5.11 ^abc^	0.25	0.21 ^a^	0.02	1.99 ^ab^	0.07
ADS	4	1.98 ^ab^	0.10	6.04 ^cd^	0.25	0.25 ^ab^	0.02	2.02 ^ab^	0.07
CF	4	1.54 ^a^	0.10	4.37 ^a^	0.25	0.23 ^ab^	0.02	1.89 ^a^	0.07
CFS	4	1.71 ^a^	0.10	5.60 ^bcd^	0.25	0.24 ^ab^	0.02	1.96 ^ab^	0.07
IF	4	1.80 ^ab^	0.10	5.70 ^bcd^	0.25	0.23 ^ab^	0.02	2.12 ^ab^	0.07
IFS	4	2.20 ^b^	0.10	6.28 ^d^	0.25	0.28 ^b^	0.02	2.24 ^b^	0.07
*F-*value		4.63		7.71		3.94		3.50	
df*		6,21		6,21		6,21		6,21	
*P-*value		0.0038		0.0002		0.0085		0.028	

Means and standard errors are shown (*n =* 4). Different letters within each column indicate significant differences among treatments (Tukey’s, *p <* 0.05). F-values, degrees of freedom (*df*; factor, error) and *p*-values are shown.

### 3.3 Detection of fecal indicator bacteria and *Salmonella*

Although fecal indicator bacteria (coliforms and *E. coli*) and *Salmonella* were detected in the digestate material, they were not detected in lettuce leaves across treatments. Coliforms and *E. coli* remained below the detection limit (<3 MPN/g), while *Salmonella* spp. was not detected by the culture-based presence/absence method employed. Detailed results are provided in Supplementary Table S4.

### 3.4 Abundance of antibiotic resistance genes and integrons

The abundance of ARGs varied across treatments but did not consistently increase in digestate fertilized plants compared with inorganic fertilization or the control. For *tetW* (*p* = 0.0252, Table 4), B exhibited the highest mean abundance, significantly higher than ADS, while all other treatments showed intermediate values and did not differ significantly from one or both extremes. A similar pattern was found for the *ermB* gene (*p* = 0.0215, Table 4), where the highest abundance was observed in B. In this case, CF showed the lowest abundance and all other treatments exhibited intermediate values. For *bla*_CTX_, differences among treatments were also statistically significant (*p* = 0.0061), with ADS showing the lowest mean, similar than with *tetW* (Table 4). On the contrary, no significant differences among treatments were observed for MGEs. Relative abundances showed the same overall trends as those observed for absolute abundances (Supplementary Table S5).

**Table 4:** Mean values of absolute abundance [log_10_ (gene copies μg^−1^ DNA)].

Treatment	*n*	*16 s*		*tetW*		*ermB*		*bla_CTX_*		*intI1*		*aint1*	
		Mean	S. E.	Mean	S. E.	Mean	S. E.	Mean	S. E.	Mean	S. E.	Mean	S. E.
C	3	5.69 ^b^	0.24	4.86 ^ab^	0.11	6.16 ^ab^	0.22	5.22 ^b^	0.15	3.37	0.24	5.56	0.17
B	3	4.93 ^ab^	0.24	5.01 ^b^	0.11	6.46 ^b^	0.22	5.22 ^b^	0.15	4.24	0.24	5.09	0.17
AD	3	5.55 ^b^	0.24	4.72 ^ab^	0.11	5.94 ^ab^	0.22	5.18 ^ab^	0.15	3.16	0.24	5.33	0.17
ADS	3	6.10 ^b^	0.24	4.40 ^a^	0.11	5.41 ^ab^	0.22	4.45 ^a^	0.15	4.09	0.24	5.10	0.17
CF	3	5.89 ^b^	0.24	4.85 ^ab^	0.11	5.34 ^a^	0.22	4.96 ^ab^	0.15	3.89	0.24	5.11	0.17
CFS	3	6.06 ^b^	0.24	4.57 ^ab^	0.11	5.58 ^ab^	0.22	4.53 ^ab^	0.15	4.31	0.24	5.07	0.17
IF	3	5.47 ^b^	0.24	4.58 ^ab^	0.11	5.79 ^ab^	0.22	5.23 ^b^	0.15	4.09	0.24	5.09	0.17
IFS	3	4.27 ^a^	0.24	4.56 ^ab^	0.11	5.39 ^ab^	0.22	4.84 ^ab^	0.15	4.22	0.24	4.64	0.17
*F-*value		6.59		3.35		3.35		4.50		2.73		2.25	
df*		7,16		7,16		7,16		7,16		7,14		7,14	
*p-*value		0.0009		0.0212		0.0215		0.0061		n.s.		n.s.	

The mean values, standard errors of the mean (S. E.) and number of observations (*n*) are indicated. *p*-values and degrees of freedom associated with the main effects are indicated at the bottom of the table. Significant *p*-values are shown, n.s. indicates no significant effect. Different lowercase letters indicate significant differences (Tukey’s, *p <* 0.05). *F*-values, degrees of freedom (*df*; factor, error) and *p*-values are shown.

### 3.5 Bacterial diversity and community composition

Statistical analyses of the alpha-diversity metrics revealed no significant effects of fertilization (Tukey’s, *p* > 0.05, Supplementary Table S6).

A beta-diversity analysis was performed to assess differences in bacterial community composition among treatments. PERMANOVA indicated that treatment explained a modest proportion of the overall variation in community composition (R^2^ = 0.461, *F* = 1.96, *p* = 0.004). However, pairwise PERMANOVA comparisons revealed no significant differences between individual treatments (p > 0.05; Supplementary Table S7). Consistent with these results, the NMDS ordination suggested only a partial separation of the control samples from the treatment samples, while no clear separation was observed among the fertilization treatments themselves (Supplementary Figure S1).

Regarding the taxonomic composition, six PCs accounted for 62.09% of the variability in the 39 most contributing ASVs. Of the six PCs obtained, the analysis focused primarily on those showing statistically significant differences among treatments (PC1, PC2 and PC5), while components without differences are briefly described for reference.

**Figure 2:**
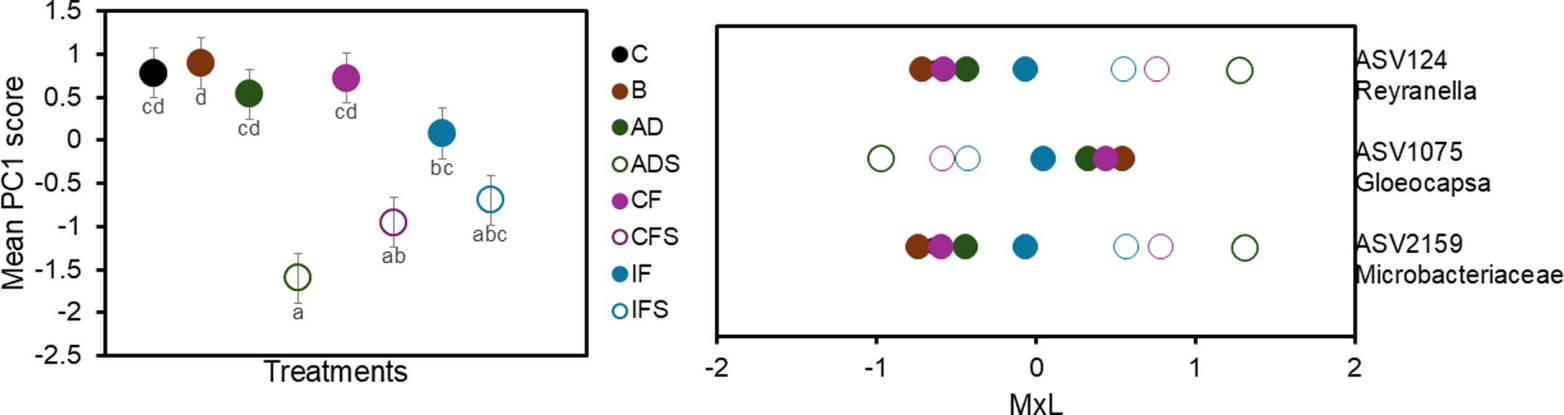
Visual representation of the combined results of the PCAs and the mean separation procedure. *Left*: Mean PC1 scores for each treatment with standard errors (error bars). Different letters indicate significant differences between treatments (Tukey’s test, *p <* 0.05). *Right:* Contribution of selected indicator ASVs (|loading| > 0.60) to the PC1 mean values for each treatment, calculated as the product of treatment means and ASV loadings (M × L).

PC1 explained 17.05% of the variability, with positive loadings for ASV731, ASV1075 and ASV1280 (genera *Microvirga, Gloeocapsa and Rhodobacter*, respectively), ASV1799 (family *Blastocatellaceae*) and ASV2044 (genus *Burkholderia-Caballeronia-Paraburkholderia*), and negative loadings for ASV124 (genus *Reyranella*), ASV868 and ASV2159 (families *Xanthomonadaceae* and *Microbacteriaceae*, respectively). PC1 showed statistically significant differences among fertilization treatments (*p <* 0.0001, Figure 2; Supplementary Table S8). C and B, together with AD and CF, presented the highest mean PC1 scores, whereas ADS and CFS showed the lowest values. Inorganic fertilization treatments exhibited intermediate scores. Certain taxa, namely *Reyranella, Microbacteriaceae, and Xanthomonadaceae*, showed positive associations with treatments involving split fertilization regimes (ADS, CFS, IFS). In contrast, *Microvirga, Gloeocapsa, Rhodobacter, Blastocatellaceae, and Burkholderia-Caballeronia-Paraburkholderia* displayed negative associations with split fertilization treatments but positive associations with single-application treatments (AD and CF), as well as with C and B. Taxa with the highest contributions (absolute loading values > 0.6) are shown in Figure 2.

**Figure 3:**
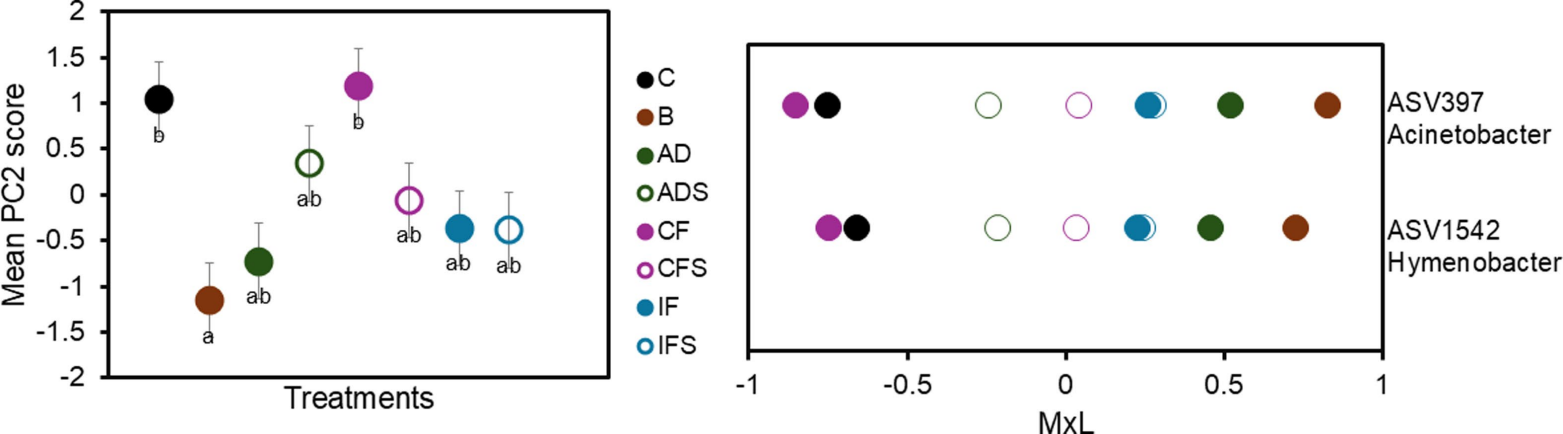
Visual representation of the combined results of the PCAs and the mean separation procedure. *Left*: Mean PC2 scores for each treatment with standard errors (error bars). Different letters indicate significant differences between treatments (Tukey’s test, *p <* 0.05). *Right:* Contribution of selected indicator ASVs (|loading| > 0.60) to the PC2 mean values for each treatment, calculated as the product of treatment means and ASV loadings (M × L).

PC2 captured 10.89% of the variability, with negative loadings for ASV397, ASV666, ASV929 and ASV1542 (genera *Acinetobacter, Brevundimonas, Bdellovibrio and Hymenobacter*, respectively), and showed statistically significant differences among fertilization treatments (*p* = 0.0142, Figure 2; Supplementary Table S8). Treatments C and CF exhibited the highest mean PC2 scores, while treatment B showed the lowest value. Based on MxL values, all taxa behaved similarly: they showed positive associations with B, AD, and both inorganic fertilization treatments, an intermediate association with CFS, and negative associations with the remaining treatments. Taxa with the highest contributions (absolute loading values > 0.6) are shown in Figure 3.

PC3 explained 10.45% of the variability and showed positive loadings for ASV1022 (order *Nostocales*) ASV1165 and ASV2170 (genera *Sphingomonas* and *Frankia*, respectively), and negative loadings for ASV735, ASV757 (genera *Allorhizobium-Neorhizobium-Pararhizobium-Rhizobium* and *Skermanella*, respectively) and ASV2057 (order *Betaproteobacteriales*). No significant differences were found among treatments (*p* = 0.0933); this PC will not be further discussed. PC4 accounted for 8.63% of the variability and exhibited a positive loading for ASV658, which belongs to the genera *Brevundimonas*, and negative loadings for ASV2000 and ASV2074 (order *Betaprotobacteriales*) and ASV2170 (genus *Frankia*). No significant differences were found among treatments (*p* = 0.1766); this PC will not be further discussed.

**Figure 4:**
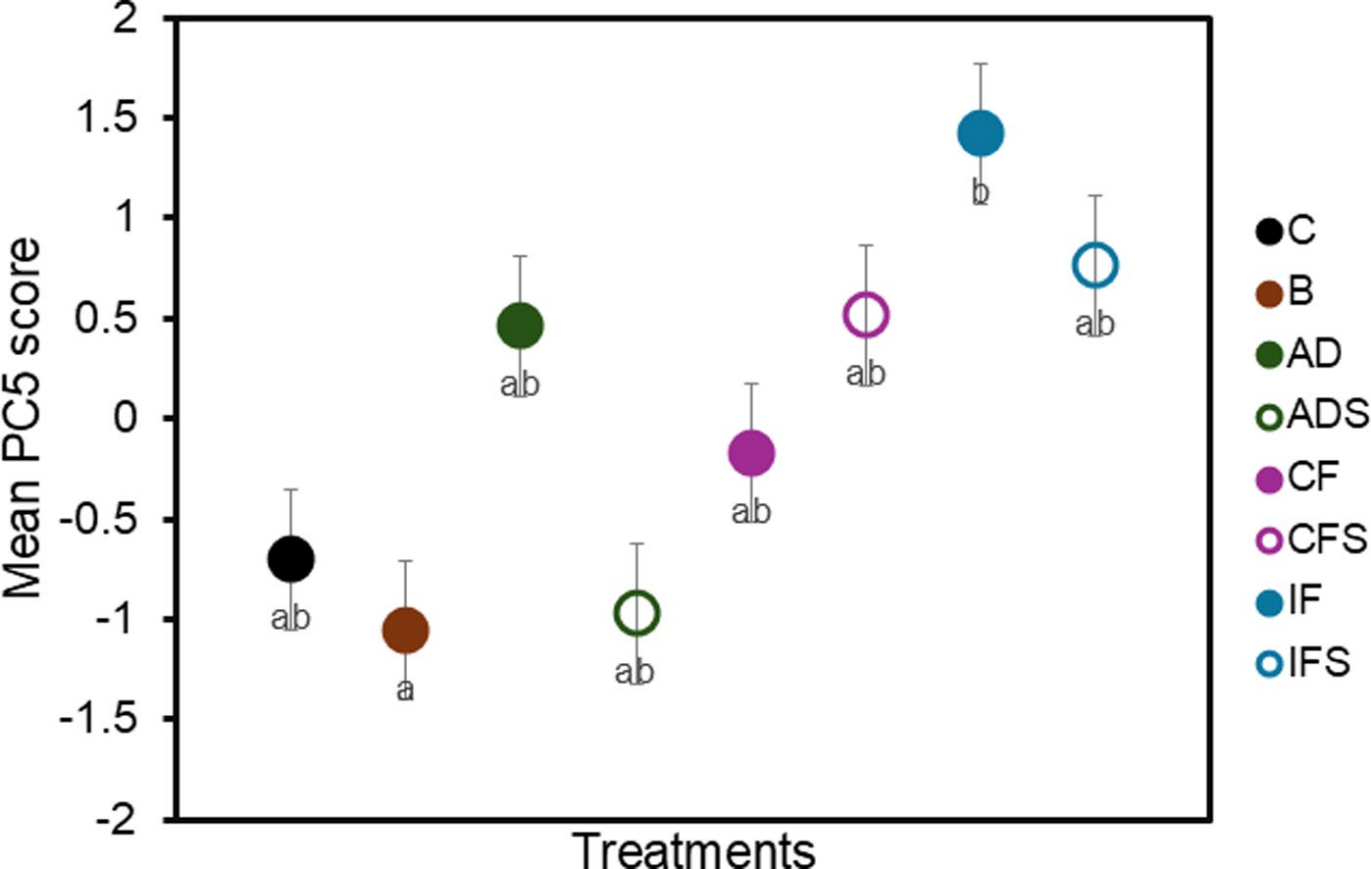
Visual representation of the PCAs. Mean values of the PC5 scores for each treatment with their standard errors (as error bars). Different letters indicate significant differences between treatments (Tukey’s, *p <* 0.05).

PC5 explained 8.44% of the variability and a positive loading for ASV793 (genus *Skermanella*). The highest PC5 scores were observed in treatment IF, which differed significantly from treatment B, the lowest-scoring group (*p* = 0.0014, Figure 4; Supplementary Table S8). The genus *Skermanella* exhibited a distinctive pattern in component 5. It showed positive associations with the two inorganic fertilizer treatments (IF and IFS), as well as with AD and CFS. The CF treatment showed no clear association, and the remaining treatments displayed negative associations. No taxa showed absolute loading values > 0.6 for this PC.

PC6 explained 6.63% of the variability and showed a positive loading for ASV825, which belongs to the genus *Pseudomonas*. No significant differences were found among treatments (*p* = 0.7244); this PC will not be further discussed.

### 3.6 Correlation analysis between plant growth parameters, nutrient composition, antibiotic resistance genes and principal components

**Figure 5:**
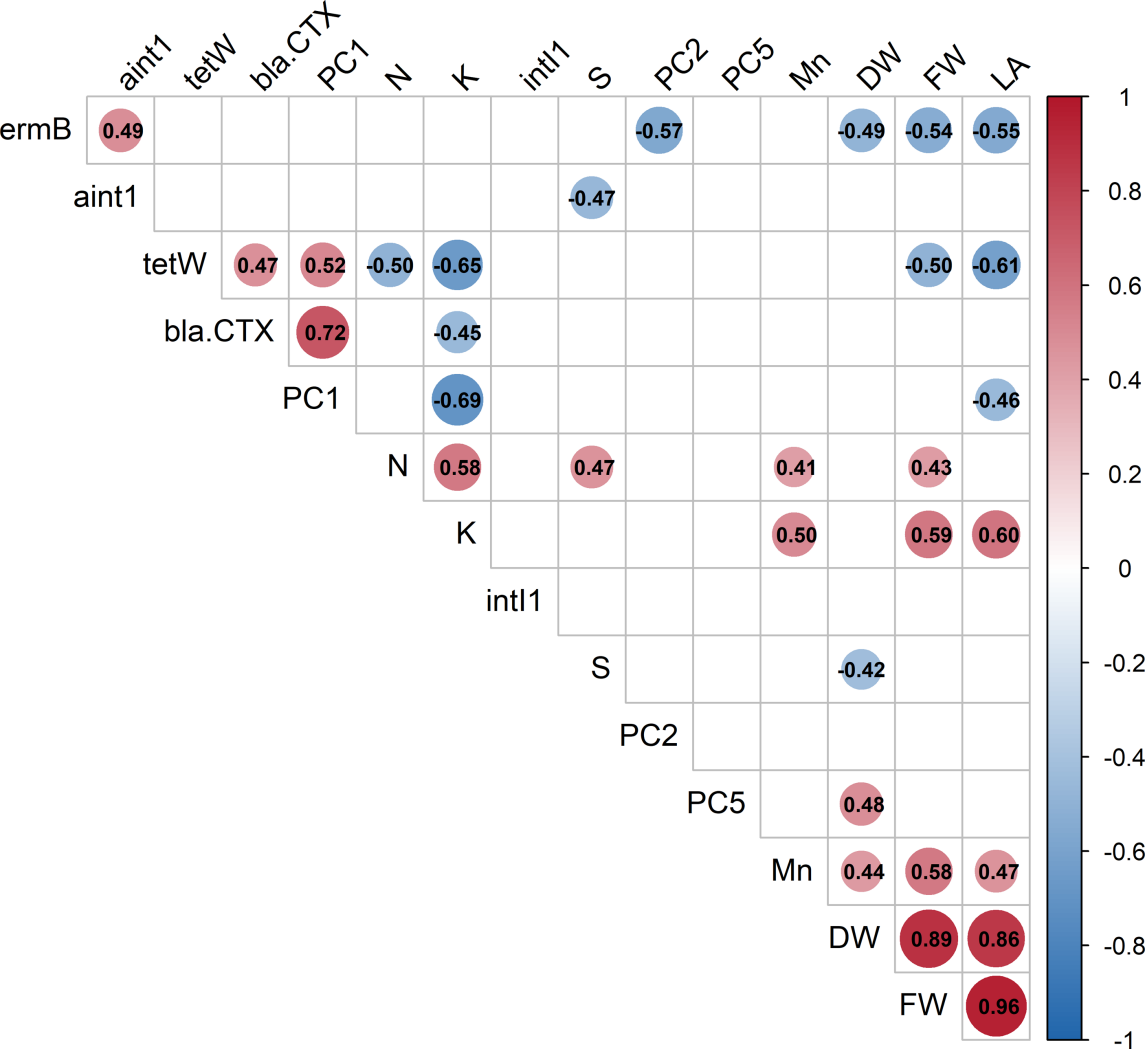
Heatmap showing Pearson correlation coefficients between plant growth parameters (FW: fresh weight, DW: dry weight, LA: leaf area), nutrients that showed significant differences (N, K, S and Mn), the relative abundance of selected ARGs (*bla*_CTX_*, ermB, tetW*) and mobile genetic elements (*intI1, aint1*), and Principal Components that showed significant differences between treatments (PC1, PC2 and PC5). Only statistically significant correlations (*p <* 0.05) with Pearson correlation coefficients |r| > 0.4 are displayed.

Pearson correlation analysis revealed association patterns among plant growth parameters, nutrient composition, ARGs, and PCs (Figure 5; Supplementary Table S9). Plant growth parameters (FW, DW and LA) were strongly and positively correlated with each other and also showed positive correlations with K and Mn. N showed a moderate positive correlation with FW, while also exhibiting positive associations with the other nutrients in the correlation matrix. *tetW* and *ermB* exhibited moderate negative correlations with plant growth parameters, particularly FW and LA, and were also negatively correlated with some nutrient variables, especially K. In contrast, *bla*_CTX_ and *intI1* showed weaker or inconsistent associations. Additionally, *bla*_CTX_ and *tetW* were positively correlated with PC1. Overall, plant growth parameters were mainly positively associated with nutrient variables, whereas some ARGs exhibited negative correlations with growth-related traits and positive associations with PC1. All Pearson correlation coefficients and corresponding *p*-values are shown in Supplementary Table S9.

## 4 Discussion

This study examined the effects of manure-derived anaerobic digestate applied to the soil, either alone or in combination with wood biochar, using *L. sativa* as a model plant. By integrating plant growth and nutritional parameters with phyllosphere bacterial community composition and selected ARG markers, fecal indicator bacteria and *Salmonella* under different fertilization strategies, our results provide insights into digestate-associated responses beyond agronomic performance.

Fertilization treatments significantly enhanced plant biomass compared to the control and biochar conditions, as reflected by higher FW and DW and increased LA (Table 2). Split inorganic fertilization resulted in the highest biomass production, likely due to the continuous and synchronized availability of readily assimilable N. Treatments with digestate achieved similar values to the single-dose inorganic fertilization treatment, suggesting that digestate supplied sufficient nutrients to sustain plant growth, although nutrient released from these organic sources may have been more gradual and less synchronized with crop demand than that from the split dose. The lower biomass observed in some digestate-biochar treatments may additionally reflect nutrient immobilization or reduced nutrient availability associated with biochar addition. These findings agree with previous studies showing the fertilizing potential of manure-based digestates across different crops. For example, Valentinuzzi et al. (2020) showed that both liquid and pelleted digestates supported biomass production in cucumber and maize, with plant responses dependent on crop species and application rate. Similarly, Fedeli et al. (2023) reported increased yield and improved quality in horticultural crops following digestate application, while Tshikalange et al. (2022) observed comparable biomass production in spinach fertilized with manure digestate and mineral fertilizer. Together, these studies and our results support the idea that digestates can function effectively as fertilizers, providing sufficient nutrient availability to sustain plant biomass production, with plant responses shaped by management practices and fertilizer type, among other factors.

As expected, plant growth responses were accompanied by changes in nutrient composition, particularly for elements closely linked to growth and metabolic activity. Although IFS generally promoted greater nutrient accumulation, ADS reached comparable values (Table 3). Overall, this pattern suggests that digestate applications supplied enough of these nutrients or improved their availability through enhanced soil nutrient mobilization. The fact that digestate provided nutrient amounts comparable to inorganic fertilization is especially relevant, given that these elements play key roles in plant physiology. Adequate N and K are essential for photosynthesis, protein synthesis, and osmotic regulation, while S and Mn are involved in enzymatic activity and redox processes that support active growth (White and Brown, 2010). In this context, the nutrient contents observed are consistent with the physiological demands associated with increased biomass production. Although a non-fertilized control was not analyzed for nutrient content, the inorganic fertilization treatment represents the conventional agronomic practice for this crop, resembling a realistic production scenario. These results support the potential of digestate as a nutrient source for greenhouse lettuce production, contributing to circular nutrient management strategies that recycle organic waste streams.

The potential presence of enteric microorganisms associated with manure-derived amendments represents an important consideration in this context, as manure can introduce microorganisms that survive the anaerobic digestion process and can be transferred to soils and, eventually, to plant tissues. Some studies have reported that microorganisms present in soil and amendments, including *E. coli* and *Salmonella*, can reach leaf surfaces under certain conditions, particularly following organic fertilization, and even persist on plant tissues (Barra Caracciolo et al., 2022; Fabiani et al., 2025). In the present study, *E. coli* and *Salmonella* were assessed as first-level indicators of hygienic quality and potential contamination, in line with common regulatory and agronomic practices. Although both *E. coli* and *Salmonella* were detected in the initial digestate, with *Salmonella* concentrations exceeding the established regulatory limit, neither microorganism was detected on lettuce leaves at harvest. While this represents an important finding, it should not be interpreted as sufficient evidence to support the agricultural use of digestate products, particularly when they do not comply with established microbiological standards. These findings are consistent with previous studies showing that the phyllosphere represents a challenging environment for enteric bacteria. Leaf surfaces are exposed to UV radiation, fluctuating humidity and limited nutrient availability (Melotto et al., 2014). Experimental studies with *Salmonella* and *E. coli* on leafy vegetables have similarly reported that their survival is often limited and strongly influenced by environmental conditions and interactions with resident microbiota (López-Gálvez et al., 2021; Lim et al., 2014). Furthermore, Martínez-Vaz et al. (2014) demonstrated that association with the phyllosphere induces stress-response pathways related to nutrient limitation and oxidative stress, highlighting the physiological adaptations required for persistence in this habitat. It is important to note that the non-detection of coliforms, *E. coli* and *Salmonella* at harvest should be interpreted within the conditions evaluated in the present study and does not exclude the presence of stressed or viable but non-cultivable microorganisms, nor the possibility of different outcomes under other environmental conditions. Future studies incorporating temporal monitoring of soil and phyllosphere bacterial populations following digestate application would help clarify the dynamics of *E. coli* and *Salmonella* transfer, persistence, and potential dissemination throughout the crop growth cycle.

Beyond culturable pathogens, the presence and dynamics of ARGs represent an additional dimension of microbiological risk, as antibiotics administered to livestock are often excreted in feces and have been associated with the occurrence and persistence of ARGs in anaerobic digestion byproducts, soils, and plants (Barra Caracciolo et al., 2022; Cao et al., 2023; Fabiani et al., 2025; Wolak et al., 2023). Our results did not show a consistent increase in the abundance of the selected ARG markers in the lettuce phyllosphere after digestate-based treatment compared with inorganic fertilization or control treatments under the studied conditions. Furthermore, changes in these ARG abundances did not follow a gene-specific enrichment pattern. The split application of digestate consistently showed lower ARG abundances (Table 4). Although the mechanisms underlying these patterns remain unclear, they may be associated with indirect plant- or microbiome-mediated effects influencing phyllosphere microbial communities (Martín-Cardoso and San Segundo, 2025; Vorholt, 2012; Xiang et al., 2024). Nutrient availability may also influence microbial competition within the phyllosphere, where increased competition can reduce the selective advantage of carrying resistance determinants because of their associated fitness costs (Bottery et al., 2021). Under this framework, we hypothesize that the more complete nutrient supply provided by digestate could promote ecological conditions that are less favorable for the persistence of certain ARGs. Nevertheless, further studies are needed to evaluate this hypothesis and the mechanisms involved. Moreover, for *ermB*, the single application of combined fertilizer resulted in the lowest gene abundance, approximately two orders of magnitude lower than in C and B. While the reasons for this pattern remain unclear, it may reflect differences associated with amendment type and application mode, which deserve further investigation.

Importantly, the observed differences in ARG abundance were not accompanied by variations in total bacterial abundance (16S rRNA gene, Table 4), suggesting that differences in ARG abundance were not solely associated with variations in overall bacterial abundance and may instead reflect changes in community composition or microbial interactions, although these processes were not directly assessed. This interpretation is consistent with the phyllosphere being a nutrient-limited and highly variable habitat, where microbial assemblages are strongly shaped by host traits and environmental constraints (Leveau, 2019; Rangel and Leveau, 2024).

Regarding MGEs, two integrons (*intI1* and *aint1*) were evaluated in this study because of their widespread use as indicators of anthropogenic impact and their recognized role in the acquisition and dissemination of ARGs in these environments (Gillings, 2014). No significant differences were detected among treatments. However, these results should be interpreted within the scope of the selected markers, as other MGEs and horizontal gene transfer mechanisms not assessed here may also contribute to gene transfer dynamics in the phyllosphere.

Within the scope of the evaluated fecal indicator bacteria, *Salmonella,* and ARG markers, our findings suggest that management practices may influence the balance between agronomic performance and microbiological profiles associated with digestate application, although broader conclusions regarding food safety or microbiological risk should be drawn cautiously and within the context of this study.

Principal component analysis based on bacterial composition further supported the observed patterns. The main axis of variation (PC1) separated split fertilization treatments from single-application treatments and was driven by a limited number of taxa with high loadings, suggesting a gradient in resource availability rather than a pronounced shift in overall community composition. The positive association of split treatments with the alphaproteobacteria *Reyranella* and members of the *Microbacteriaceae* family suggests that more stable nutrient delivery may reflect the establishment of taxa under more sustained nutrient inputs, whereas the association of single-application treatments with the cyanobacteria *Gloeocapsa* may reflect its tolerance to greater temporal variability in nutrient inputs on the leaf surface (Figure 2, Samsri et al., 2025; Vorholt, 2012; Whitton and Potts, 2012). In contrast, PC2, driven by *Acinetobacter* and *Hymenobacter*, did not show a consistent relationship with fertilization strategy, suggesting that this axis captured taxon-specific or context-dependent variability rather than fertilization-related effects. Both genera are common phyllosphere inhabitants known for their tolerance to different environmental stressors, which may explain their contribution to this axis (Figure 3, Remus-Emsermann and Schlechter, 2018; Vorholt, 2012). Similarly, separation along PC5, particularly for inorganic fertilization treatments, was driven mainly by *Skermanella*, a soil-associated alphaproteobacterial genus frequently associated with crop rhizospheres (Choi et al., 2025), pointing to a taxon-specific response to mineral nutrient inputs rather than a broad restructuring of the community (Figure 4).

Despite these compositional shifts, alpha-diversity indices were not affected by fertilization, indicating that overall richness and evenness of the phyllosphere bacterial community remained relatively stable across treatments (Supplementary Table S6). Consistently, beta-diversity analysis showed no significant pairwise differences, suggesting that fertilization induced modest and diffuse shifts in community composition rather than the formation of distinct treatment-specific assemblages (Supplementary Table S7). Similar patterns have been reported in other studies, showing that phyllosphere-associated microbial communities often exhibit resistance of both alpha- and beta-diversity to fertilization, as host-related factors and the open, highly dynamic nature of the phyllosphere can attenuate the effects of management practices on microbial community structure (Remus-Emsermann and Schlechter, 2018; Xiong et al., 2021; Zhu et al., 2022).

Correlation analysis was used to explore associations among plant performance and nutrient contents, multivariate principal components, and ARG abundance in the phyllosphere. Plant growth parameters were strongly and positively correlated with nutrient content, especially with K and Mn, confirming that improved growth was associated with enhanced nutritional status (Figure 5; Supplementary Table S9). In contrast, *tetW* and *ermB* showed negative correlations with plant growth variables, particularly with FW and LA, and were also negatively correlated with K, suggesting that improved plant performance may affect ARG persistence indirectly, through changes mediated by the plant or its associated habitat, rather than by direct microbial enrichment. This pattern was supported by multivariate analysis. PC1 was positively correlated with *bla*_CTX_ and *tetW* and negatively correlated with K, and plant growth parameters (LA), indicating that this axis captured variations in nutrient delivery patterns arising from different fertilization practices, which were associated with higher ARG abundance but lower plant performance (Figure 5; Supplementary Table S9). In contrast, no correlations were detected between MGEs and plant growth or nutrient variables; however, this result should be interpreted cautiously, as only two MGE markers were evaluated, limiting the ability to extrapolate conclusions regarding horizontal gene transfer drivers under the studied conditions. PC5 showed positive correlations with DW, which could indicate a specific association between *Skermanella* and higher plant performance, consistent with their potential role as plant-growth promoting bacteria (Choi et al., 2025), with no corresponding relationship to ARG-related community patterns (Figure 5; Supplementary Table S9). Taken together, our findings suggest that the abundance patterns of the evaluated ARG markers in the phyllosphere were more closely associated with plant condition and microbial community composition than with direct fertilization effects or enhanced horizontal gene transfer. This interpretation is consistent with the strong host and environmental filtering characteristic of the phyllosphere discussed above, as well as with evidence that plant physiological traits can indirectly shape resistomes by influencing microbial assemblages (Rangel and Leveau, 2024; Xiang et al., 2024).

Overall, the findings contribute to a broader understanding of the interactions between digestate-based fertilization practices, plant-associated microbial communities, and resistance markers (ARGs and MGEs) in the phyllosphere. Integrating these dimensions is relevant for improving the interpretation of how nutrient management strategies may influence microbial community structure and resistance-related dynamics within circular agricultural systems.

## 5 Conclusion

This study evaluated the agronomic and microbiological implications of using digestate-based fertilizers in greenhouse lettuce production. Digestate treatments supported lettuce growth and biomass production at levels comparable to conventional inorganic fertilization, indicating that digestate can serve as an effective nutrient source in this system. While the fertilization strategy resulted in detectable shifts in the composition of phyllosphere bacterial communities, overall microbial diversity remained relatively stable across treatments. Importantly, although fecal indicator bacteria and *Salmonella* were detected in the digestate material, with *Salmonella* concentrations exceeding the established regulatory threshold, these microorganisms were not detected in lettuce leaves under the conditions and methodologies applied in this study. Similarly, the abundance of the selected ARGs varied among treatments but did not show a consistent increase in response to digestate-based fertilization. These findings should be interpreted within the context of the present study and do not warrant the safety of digestate products for agricultural use, nor do they exempt them from meeting established regulatory standards.

Taken together, our results highlight the importance of evaluating digestate use through an integrated framework that considers agronomic performance alongside microbiological and antimicrobial resistance-related parameters. Further research will be valuable for improving our understanding of the broader implications of digestate use in horticultural production systems and its role within circular nutrient management strategies.

### 5.1 Limitations and future perspectives

This study was conducted under greenhouse conditions using lettuce as a model plant, which may not fully reflect the environmental variability and complexity of field agroecosystems, including environmental factors that may influence microbial transmission and persistence. Therefore, the extrapolation of these findings to open-field production systems should be made cautiously.

While the present study focused on the harvest stage, future research incorporating intermediate sampling points and longer-term evaluations could provide additional insights into the temporal dynamics of microbial communities and antimicrobial resistance-related parameters associated with digestate-based fertilization.

Furthermore, microbiological analyses were based on standardized culture-dependent methodologies commonly used in agricultural and food safety assessments. Consequently, viable but non-culturable or dormant microorganisms could not be specifically evaluated and therefore cannot be completely excluded. Other microbial taxa of potential relevance, including opportunistic pathogens were not specifically investigated in this study. In particular, phytopathogenic microorganisms and other crop-health-related microbial groups were not included among the microbial groups evaluated in this study. Therefore, our findings do not allow conclusions regarding their response to digestate application or the potential implications for crop health and disease development. Moreover, microbiological characterization focused on selected indicator microorganisms, *Salmonella*, ARGs, and MGEs and was not intended to represent a comprehensive assessment of all potential microbial hazards associated with digestate-based fertilization. Therefore, the findings presented here should be interpreted within the context of the microbial groups evaluated. Our results provide a basis for future studies addressing a broader range of microbial taxa, ecological functions, and potential microbiological risks associated with digestate-based fertilization.

Finally, although this study provides insights into compositional microbial responses, the proposed mechanisms underlying these interactions are based on association-based analyses and require further investigation. Future studies could explore functional responses, including gene expression related to metabolic pathways, antimicrobial resistance activity, and plant-microbe signaling in the phyllosphere, to better understand their implications for plant health, ecophysiology and agroecosystem functioning.

## Data Availability

The datasets presented in this study can be found in online repositories. The names of the repository/repositories and accession number(s) can be found below: https://www.ncbi.nlm.nih.gov/, PRJNA1436558.
