# Supplementary material for: Digestate-based fertilization supports lettuce growth with limited impacts on phyllosphere bacterial communities
Source: Front Microbiol. 2026 Jul 29;17:1888705. doi: 10.3389/fmicb.2026.1888705 (PMC13461580; doi:10.3389/fmicb.2026.1888705)
Supplement: Supplementary file 1 [file Supplementary_file_1.docx]

Supplementary Material

## 1. Supplementary Tables

**Supplementary Table 1.** Chemical and spectroscopy characterization of digestate, biochar, and combined fertilizer. ND: not determined.

|  |  | **Digestate** | **Biochar** | **Combined fertilizer** |
| --- | --- | --- | --- | --- |
|  |  | **Mean ± S.E.** | **Mean ± S.E.** | **Mean ± S.E.** |
| **TS** | % | 6.15 ± 0.004 | ND* | ND |
| **VS** |  | 61.06 ± 0.004 | ND | ND |
| **C** |  | 30.43 ± 0.127 | 64.52 ± 2.3103 | ND |
| **TKN** |  | 6.73 ± 0.166 | 1.45 ± 0.0210 | 3.47 ± 0.023 |
| **N-NH4+** |  | 4.95 ± 0.100 | 0.001 ± 0.0002 | 0.621 ± 0.013 |
| **N-NO3-** |  | 0.07 ± 0.001 | 0.0008 ± 0.0002 | 1.097 ± 0.049 |
| **TP** | mg kg^-1^ | 9790 | 665 | 4673 |
| **AP** |  | 903 | 11.6 | 1215 |
| **As** |  | 0,78 | < 0.5 | < 0.5 |
| **Cd** |  | < 0.1 | < 0.1 | < 0.1 |
| **Cr** |  | 9.1 | 1.1 | 5.3 |
| **Cu** |  | 59.6 | 4.6 | 28.3 |
| **Hg** |  | < 0.1 | < 0.1 | < 0.1 |
| **Ni** |  | 8.5 | 1.2 | 2.4 |
| **Pb** |  | 1.5 | 0.61 | 0.35 |
| **Zn** |  | 334 | 16.7 | 148 |

**Supplementary Table 2**. Primers used in this study to quantify total bacteria (16s rRNA), resistance genes (*tetW, ermB, bla*_CTX_), integrons (*intI1*) and anthropogenic class 1 integrons (*aint1*).

| **Target** | **Primer** | **Sequence (5′–3′)** | **Amplicon size (bp)** | **Annealing (◦C)** | **Reference** |
| --- | --- | --- | --- | --- | --- |
| *16S rRNA* | 338_F 518_R | ACTCCTACGGGAGGCAGCAG ATTACCGCGGCTGCTGG | ~180 | 53 | Pollock et al., 2020 |
| *tetW* | tetW_F tetW_R | GAGAGCCTGCTATATGCCAGC GGGCGTATCCACAATGTTAAC | 168 | 65 | Aminov et al., 2004 |
| *ermB* | ermB_F ermB_R | AGCCATGCGTCTGACATCTA CTGTGGTATGGCGGGTAAGT | 193 | 56 | Szczepanowski et al., 2009 |
| *bla_CTX_* | RTCTX-M-F RTCTX-M-R | CTATGGCACCACCAACGATA ACGGCTTTCTGCCTTAGGTT | 101 | 61 | Marti et al., 2013 |
| *intI1* | intI1_LC1 F intI1_LC5 R | GCCTTGATGTTACCCGAGAG GATCGGTCGAATGCGTGT | 196 | 60 | Barraud et al., 2010 |
| *aint1* | aint1_F aint1_R | GCACTAAGCACATAATTG CCAACTATTGCGATAACA | 140 | 50 | Quintela-Baluja et al., 2021 |

**Supplementary Table 3.** Concentrations of nutrients in lettuce plants grown under the different fertilization treatments. Means ± standard errors are shown for variables analyzed by ANOVA and Tukey’s post-hoc test, whereas medians and interquartile ranges (IQR) are shown for variables analyzed using the Kruskal–Wallis test followed by Dunn–Bonferroni post-hoc comparisons (n = 4). No significant differences among treatments were detected for any variable.

| **Treatment** | **P (%)** | | **Mg (%)** | | **Ca (%)** | | **B (ppm)** | | **Fe (ppm)** | | **Al (ppm)** | | **Cu (ppm)** | | **Zn (ppm)** | | **Na (ppm)** | |
| --- | --- | --- | --- | --- | --- | --- | --- | --- | --- | --- | --- | --- | --- | --- | --- | --- | --- | --- |
|  | **Mean** | **S.E.** | **Mean** | **S.E.** | **Mean** | **S.E.** | **Mean** | **S.E.** | **Mean (log_10_)** | **S.E.** | **Mean (log_10_)** | **S.E.** | **Mean** | **S.E.** | **Median** | **IQR** | **Median** | **IQR** |
| **B** | 0.41 | 0.03 | 0.36 | 0.02 | 1.75 | 0.15 | 121.4 | 7.44 | 6.23 | 0.22 | 6.16 | 0.19 | 5.75 | 0.53 | 41.2 | 2.1 | 15726.2 | 1076.1 |
| **AD** | 0.34 | 0.03 | 0.31 | 0.02 | 1.44 | 0.15 | 96.2 | 7.44 | 6.22 | 0.22 | 6.17 | 0.19 | 4.45 | 0.53 | 35.5 | 5.6 | 13434.2 | 1962.3 |
| **ADS** | 0.45 | 0.03 | 0.32 | 0.02 | 1.45 | 0.15 | 100.1 | 7.44 | 6.18 | 0.22 | 6.02 | 0.19 | 6.68 | 0.53 | 41.3 | 11.2 | 15706.1 | 2867.2 |
| **CF** | 0.34 | 0.03 | 0.28 | 0.02 | 1.27 | 0.15 | 90.8 | 7.44 | 6.25 | 0.22 | 6.28 | 0.19 | 4.32 | 0.53 | 32.0 | 4.3 | 12674.3 | 3264.6 |
| **CFS** | 0.37 | 0.03 | 0.31 | 0.02 | 1.59 | 0.15 | 95.5 | 7.44 | 6.32 | 0.22 | 6.23 | 0.19 | 5.35 | 0.53 | 38.2 | 1.7 | 16650.2 | 1310.2 |
| **IF** | 0.33 | 0.03 | 0.36 | 0.02 | 1.55 | 0.15 | 83.4 | 7.44 | 6.24 | 0.22 | 6.12 | 0.19 | 4.42 | 0.53 | 36.2 | 3.7 | 14215.8 | 1319.7 |
| **IFS** | 0.33 | 0.03 | 0.34 | 0.02 | 1.62 | 0.15 | 90.2 | 7.44 | 6.52 | 0.22 | 6.41 | 0.19 | 6.08 | 0.53 | 63.6 | 18.4 | 15756.2 | 1518.2 |

**Supplementary Table 4.** Microbiological quality of plant samples under different treatments. Values correspond to the most probable number (MPN/g) of total coliforms, fecal coliforms and *E. Coli* with corresponding confidence intervals (CI). Detection limit <3 MPN g⁻¹. *Salmonella* was not detected in 20 g.

|  | **Coliforms** | | | | |  | ***Salmonella*** |
| --- | --- | --- | --- | --- | --- | --- | --- |
|  | **Total coliforms** | | **Fecal coliforms** | | ***E. coli*** |  |  |
|  | **MPN/g** | **CI** | **MPN/g** | **CI** | **MPN/g** | **CI** |  |
| **C** | < 3 | 0-9.5 | < 3 | 0-9.5 | < 3 | 0-9.5 | Absence |
| **AD** | < 3 | 0-9.5 | < 3 | 0-9.5 | < 3 | 0-9.5 | Absence |
| **ADS** | < 3 | 0-9.5 | < 3 | 0-9.5 | < 3 | 0-9.5 | Absence |
| **CF** | < 3 | 0-9.5 | < 3 | 0-9.5 | < 3 | 0-9.5 | Absence |
| **CFS** | < 3 | 0-9.5 | < 3 | 0-9.5 | < 3 | 0-9.5 | Absence |
| **IF** | < 3 | 0-9.5 | < 3 | 0-9.5 | < 3 | 0-9.5 | Absence |

**Supplementary Table 5.** Mean values of relative abundance [log_10_ (target gene copies µg^−1^ DNA/ 16S rRNA gene copies µg^−1^ DNA)]. The mean values, standard errors of the mean (S.E.) and number of observations (n) are indicated. P-values and degrees of freedom associated with the main effects are indicated at the bottom of the table. Significant p-values are shown, n.s. indicates no significant effect. Different lowercase letters indicate significant differences (Tukey’s, p<0.05). Degrees of freedom (*df*) are shown.

| **Gene / integron** | | ***tetW*** | | ***ermB*** | | ***bla*_CTX_** | | ***intI1*** | | ***aint1*** | |
| --- | --- | --- | --- | --- | --- | --- | --- | --- | --- | --- | --- |
| **Treatment** | **n** | **Mean** | **S.E.** | **Mean** | **S.E.** | **Mean** | **S.E.** | **Mean** | **S.E.** | **Mean** | **S.E.** |
| **C** | 3 | 1.60E-4 | 1.90E-3 | 0.0037 _ab_ | 0.01 | 3.40E-04 | 2.10E-3 | 5.60E-6 | 5.40E-4 | 1.00E-3 | 1.10E-3 |
| **B** | 3 | 0.01 | 1.90E-3 | 0.04 _a_ | 0.01 | 0.01 | 2.10E-3 | 2.60E-4 | 5.40E-4 | 2.50E-3 | 1.10E-3 |
| **AD** | 3 | 2.00E-4 | 1.90E-3 | 0.01 _a_ | 0.01 | 8.20E-04 | 2.10E-3 | 1.00E-5 | 5.40E-4 | 4.30E-4 | 1.10E-3 |
| **ADS** | 3 | 2.50E-5 | 1.90E-3 | 2.70E-4 _a_ | 0.01 | 2.50E-05 | 2.10E-3 | 1.20E-5 | 5.40E-4 | 1.90E-4 | 1.10E-3 |
| **CF** | 3 | 1.10E-4 | 1.90E-3 | 3.50E-4 _a_ | 0.01 | 1.30E-04 | 2.10E-3 | 1.20E-5 | 5.40E-4 | 1.80E-4 | 1.10E-3 |
| **CFS** | 3 | 3.30E-5 | 1.90E-3 | 3.20E-4 _a_ | 0.01 | 3.60E-05 | 2.10E-3 | 2.50E-5 | 5.40E-4 | 1.00E-4 | 1.10E-3 |
| **IF** | 3 | 1.50E-4 | 1.90E-3 | 2.10E-3 _a_ | 0.01 | 8.20E-04 | 2.10E-3 | 4.50E-5 | 5.40E-4 | 1.30E-3 | 1.10E-3 |
| **IFS** | 3 | 3.10E-3 | 1.90E-3 | 0.02 _ab_ | 0.01 | 0.01 | 2.10E-3 | 2.60E-3 | 5.40E-4 | 4.40E-3 | 1.10E-3 |
| **7 df** |  |  |  |  |  |  |  |  |  |  |  |
| ***p-*values** |  | n.s | | 0.007 | | n.s | | n.s | | n.s | |

**Supplementary Table 6.** Treatment mean values (mean), standard errors of the mean (S.E.), number of observations (n), as well as probability values (p-value), and degrees of freedom (df) associated with the ANOVA alpha-diversity metrics of the S, observed richness; H’, Shannon index; Chao-1 index; J, Pielou's evenness; and 1/D, inverse Simpson index. Degrees of freedom and p-values are shown.

|  |  | **S** |  | **H’** | | **Chao-1** | | **J** | | **1/D** | |
| --- | --- | --- | --- | --- | --- | --- | --- | --- | --- | --- | --- |
| **Treatment** | **n** | **Mean** | **S.E.** | **Mean** | **S.E.** | **Mean** | **S.E.** | **Mean** | **S.E.** | **Mean** | **S.E.** |
| **C** | 3 | 146.00 | 31.64 | 3.04 | 0.36 | 164.08 | 38.78 | 0.61 | 0.05 | 11.25 | 2.58 |
| **B** | 3 | 118.00 | 31.64 | 2.73 | 0.36 | 143.82 | 38.78 | 0.58 | 0.05 | 6.85 | 2.58 |
| **AD** | 3 | 154.66 | 31.64 | 2.96 | 0.36 | 178.77 | 38.78 | 0.59 | 0.05 | 7.49 | 2.58 |
| **ADS** | 3 | 153.33 | 31.64 | 3.11 | 0.36 | 215.66 | 38.78 | 0.61 | 0.05 | 9.59 | 2.58 |
| **CF** | 3 | 157.33 | 31.64 | 2.92 | 0.36 | 198.16 | 38.78 | 0.57 | 0.05 | 7.76 | 2.58 |
| **CFS** | 3 | 134.33 | 31.64 | 2.98 | 0.36 | 164.64 | 38.78 | 0.61 | 0.05 | 8.86 | 2.58 |
| **IF** | 3 | 137.33 | 31.64 | 2.80 | 0.36 | 162.39 | 38.78 | 0.57 | 0.05 | 6.41 | 2.58 |
| **IFS** | 3 | 71.33 | 31.64 | 1.77 | 0.36 | 77.90 | 38.78 | 0.40 | 0.05 | 3.45 | 2.58 |
|  | **df** | ***p*-values** | | | | | | | | | |
|  | 7 | 0.591 | | 0.248 | | 0.393 | | 0.183 | | 0.588 | |

**Supplementary Table 7.** Pairwise PERMANOVA comparisons between treatment groups based on weighted UniFrac distances (1000 permutations). P-values were adjusted for multiple comparisons using the false discovery rate (FDR) method. No comparisons were statistically significant at p < 0.05.

|  | **C** | **B** | **AD** | **ADS** | **CF** | **CFS** | **IF** | **IFS** |
| --- | --- | --- | --- | --- | --- | --- | --- | --- |
| **C** | 1 | - | - | - | - | - | - |  |
| **B** | 0.22 | 1 | - | - | - | - | - |  |
| **AD** | 0.22 | 0.86 | 1 | - | - | - | - |  |
| **ADS** | 0.22 | 0.90 | 0.73 | 1 | - | - | - |  |
| **CF** | 0.22 | 0.73 | 0.56 | 0.73 | 1 | - | - |  |
| **CFS** | 0.22 | 0.44 | 0.22 | 0.82 | 0.40 | 1 | - |  |
| **IF** | 0.22 | 0.90 | 0.86 | 0.44 | 0.44 | 0.22 | 1 |  |
| **IFS** | 0.22 | 0.22 | 0.44 | 0.22 | 0.22 | 0.22 | 0.44 | 1 |

**Supplementary Table 8.** Mean and S.E. scores of the three principal components (PC) that showed significant differences among treatments. Different letters within each PC indicate significant differences among treatments (Tukey’s, p < 0.05). F-values, degrees of freedom (df; factor, error) and p-values are shown.

| **Treatment** | **PC1** | | **PC2** | | **PC5** | |
| --- | --- | --- | --- | --- | --- | --- |
|  | **Mean** | **S.E.** | **Mean** | **S.E.** | **Mean** | **S.E.** |
| **C** | 0.78 ^cd^ | 0.29 | 1.04 ^b^ | 0.41 | -0.70 ^ab^ | 0.35 |
| **B** | 0.89 ^d^ | 0.29 | -1.15 ^a^ | 0.41 | -1.05 ^b^ | 0.35 |
| **AD** | 0.53 ^cd^ | 0.29 | -0.76 ^ab^ | 0.41 | 0.46 ^ab^ | 0.35 |
| **ADS** | -1.60 ^a^ | 0.29 | 0.33 ^ab^ | 0.41 | -0.97 ^ab^ | 0.35 |
| **CF** | 0.72 ^cd^ | 0.29 | 1.18 ^b^ | 0.41 | -0.17 ^ab^ | 0.35 |
| **CFS** | -0.95 ^ab^ | 0.29 | -0.05 ^ab^ | 0.41 | 0.51 ^ab^ | 0.35 |
| **IF** | 0.07 ^bc^ | 0.29 | -0.36 ^ab^ | 0.41 | 1.42 ^a^ | 0.35 |
| **IFS** | -0.69 ^abc^ | 0.36 | -0.38 ^ab^ | 0.51 | 0.76 ^ab^ | 0.43 |
| ***F*** | *9.93* | | *3.80* | | *6.28* | |
| ***df**** | *7,15* | | *7,15* | | *7,15* | |
| ***p-values*** | *<0.0001* | | *0.0142* | | *0.0014* | |
|  |  | |  | |  | |

**Supplementary Table 9.** Pearson correlation coefficients (below the diagonal) and associated p-values (above the diagonal) between plant growth parameters (FW: fresh weight, DW: dry weight, LA: leaf area), nutrients that showed significant differences (N, K, S, Mn and Cu), the relative abundance of selected antibiotic resistance genes (*bla*_CTX_, *ermB, tetW*) and mobile genetic elements (*intI1, aint1*), and Principal Components that showed significant differences between treatments (PC1, PC2 and PC5).

|  | **FW** | **DW** | **LA** | ***bla*_CTX_** | ***ermB*** | ***tetw*** | ***intI1*** | ***aint1*** | **N** | **K** | **S** | **Mn** | **Cu** | **PC1** | **PC2** | **PC5** |
| --- | --- | --- | --- | --- | --- | --- | --- | --- | --- | --- | --- | --- | --- | --- | --- | --- |
| **FW** |  | 1.3e^-7^ | 4.6e^-11^ | 0.78 | 0.023 | 0.035 | 0.67 | 0.67 | 0.053 | 0.035 | 0.74 | 0.001 | 0.99 | 0.095 | 0.91 | 0.051 |
| **DW** | 0.89 |  | 1.8e^-6^ | 0.59 | 0.042 | 0.38 | 0.33 | 0.86 | 0.84 | 0.71 | 0.12 | 0.052 | 0.12 | 0.86 | 0.93 | 0.06 |
| **LA** | 0.96 | 0.86 |  | 0.39 | 0.031 | 0.011 | 0.91 | 0.14 | 0.91 | 0.039 | 0.37 | 0.017 | 0.93 | 0.074 | 0.73 | 0.11 |
| ***bla*_CTX_** | -0.07 | 0.12 | -0.20 |  | 0.28 | 0.03 | 0.67 | 0.76 | 0.49 | 0.12 | 0.95 | 0.64 | 0.15 | 0.000 | 0.25 | 0.27 |
| ***ermB*** | -0.54 | -0.49 | -0.55 | 0.25 |  | 0.16 | 0.76 | 0.029 | 0.62 | 0.55 | 0.43 | 0.18 | 0.68 | 0.067 | 0.009 | 0.23 |
| ***tetW*** | -0.50 | -0.20 | -0.61 | 0.47 | 0.32 |  | 0.4 | 0.63 | 0.069 | 0.013 | 0.63 | 0.27 | 0.21 | 0.02 | 0.56 | 0.36 |
| ***intI1*** | 0.10 | -0.23 | 0.03 | -0.10 | -0.07 | -0.20 |  | 0.75 | 0.16 | 0.17 | 0.1 | 0.1 | 0.16 | 0.16 | 0.87 | 0.82 |
| ***aint1*** | -0.10 | 0.04 | -0.03 | 0.07 | 0.49 | 0.11 | -0.08 |  | 0.93 | 0.35 | 0.049 | 0.2 | 0.98 | 0.83 | 0.94 | 0.85 |
| **N** | 0.43 | 0.05 | 0.33 | -0.16 | 0.11 | -0.41 | 0.32 | 0.02 |  | 0.003 | 0.042 | 0.013 | 0.003 | 0.12 | 0.31 | 0.78 |
| **K** | 0.59 | 0.09 | 0.60 | -0.45 | -0.14 | -0.53 | 0.32 | -0.22 | 0.58 |  | 0.054 | 0.021 | 0.029 | 0.042 | 0.34 | 0.27 |
| **S** | -0.08 | -0.42 | -0.21 | 0.02 | 0.18 | 0.11 | 0.38 | -0.47 | 0.47 | 0.43 |  | 0.033 | 0.019 | 0.55 | 0.37 | 0.5 |
| **Mn** | 0.58 | 0.44 | 0.47 | 0.11 | -0.30 | -0.25 | 0.38 | -0.30 | 0.41 | 0.50 | 0.47 |  | 0.19 | 0.32 | 0.56 | 0.14 |
| **Cu** | 0.01 | -0.35 | 0.02 | -0.33 | 0.10 | -0.29 | 0.33 | -0.01 | 0.61 | 0.48 | 0.51 | 0.30 |  | 0.051 | 0.66 | 0.33 |
| **PC1** | -0.38 | -0.04 | -0.46 | 0.72 | 0.42 | 0.52 | -0.33 | 0.05 | -0.50 | -0.69 | -0.14 | -0.23 | -0.44 |  | 0.58 | 0.7 |
| **PC2** | 0.03 | 0.20 | 0.08 | -0.27 | -0.57 | -0.14 | 0.04 | -0.02 | -0.24 | -0.23 | -0.47 | -0.14 | -0.11 | -0.13 |  | 0.61 |
| **PC5** | 0.44 | 0.48 | 0.37 | 0.26 | -0.28 | -0.22 | -0.06 | 0.05 | 0.07 | 0.26 | -0.16 | 0.34 | -0.23 | 0.09 | 0.12 |  |

2. **Supplementary Figures**

**Supplementary Figure 1.** Beta diversity visualized using non-metric multidimensional scaling (NMDS) based on weighted UniFrac distances. Each point represents a sample. Treatments are indicated by their assigned codes. Ellipses represent standard errors around group centroids. NMDS axes represent non-metric dimensions summarizing variation in community composition. Stress = 0.09157.

**References**

Aminov, R. I., Chee-Sanford, J. C., Garrigues, N., Mehboob, A., and Mackie, R. I. (2004). Detection of tetracycline resistance genes by PCR methods. Methods Mol. Biol. 268, 3–13. doi: 10.1385/1-59259-766-1:003

Barraud, O., Baclet, M. C., Denis, F., and Ploy, M. C. (2010). Quantitative multiplex realtime PCR for detecting class 1, 2 and 3 integrons. J. Antimicrob. Chemother. 65, 1642–1645. doi: 10.1093/jac/dkq167

Marti, E., Jofre, J., and Balcazar, J. L. (2013). Prevalence of antibiotic resistance genes and bacterial community composition in a river influenced by a wastewater treatment plant. PLoS One 8:e78906. doi: 10.1371/journal.pone.0078906

Pollock, J., Muwonge, A., Hutchings, M. R., Mainda, G., Bronsvoort, B. M., Gally, D., et al. (2020). Resistance to change: AMR gene dynamics on a commercial pig farm with high antimicrobial usage. Sci. Rep. 10:1708. doi: 10.1038/s41598-020-58659-3

Quintela-Baluja, M., Frigon, D., Abouelnaga, M., Jobling, K., Romalde, J. L., Gomez Lopez, M., et al. (2021). Dynamics of integron structures across a wastewater network – implications to resistance gene transfer. Water Res. 206:117720. doi: 10.1016/j. watres.2021.117720

Szczepanowski, R., Linke, B., Krahn, I., Gartemann, K. H., Gützkow, T., Eichler, W., et al. (2009). Detection of 140 clinically relevant antibiotic-resistance genes in the plasmid metagenome of wastewater treatment plant bacteria showing reduced susceptibility to selected antibiotics. Microbiology 155, 2306–2319. doi: 10.1099/mic.0.028233-0
